# A fast and accurate algorithm for single individual haplotyping

**DOI:** 10.1186/1752-0509-6-S2-S8

**Published:** 2012-12-12

**Authors:** Minzhu Xie, Jianxin Wang, Tao Jiang

**Affiliations:** 1 College of Physics and Information Science, Hunan Normal University, Changsha 410081, P. R. China; 2 School of Information Science and Engineering, Central South University, Changsha 410083, P. R. China; 3 Department of Computer Science and Engineering, University of California, Riverside, CA 92521, USA

## Abstract

**Background:**

Due to the difficulty in separating two (paternal and maternal) copies of a chromosome, most published human genome sequences only provide genotype information, *i.e*., the mixed information of the underlying two haplotypes. However, phased haplotype information is needed to completely understand complex genetic polymorphisms and to increase the power of genome-wide association studies for complex diseases. With the rapid development of DNA sequencing technologies, reconstructing a pair of haplotypes from an individual's aligned DNA fragments by computer algorithms (*i.e*., Single Individual Haplotyping) has become a practical haplotyping approach.

**Results:**

In the paper, we combine two measures "errors corrected" and "fragments cut" and propose a new optimization model, called Balanced Optimal Partition (BOP), for single individual haplotyping. The model generalizes two existing models, Minimum Error Correction (MEC) and Maximum Fragments Cut (MFC), and could be made either model by using some extreme parameter values. To solve the model, we design a heuristic dynamic programming algorithm H-BOP. By limiting the number of intermediate solutions at each iteration to an appropriately chosen small integer *k*, H-BOP is able to solve the model efficiently.

**Conclusions:**

Extensive experimental results on simulated and real data show that when *k *= 8, H-BOP is generally faster and more accurate than a recent state-of-art algorithm ReFHap in haplotype reconstruction. The running time of H-BOP is linearly dependent on some of the key parameters controlling the input size and H-BOP scales well to large input data. The code of H-BOP is available to the public for free upon request to the corresponding author.

## Background

Each human somatic cell contains 23 pairs of chromosomes, and there are about 0.5% differences between the DNA sequences of two copies of each chromosome [1]. The dominant DNA differences are single nucleotide polymorphisms (SNPs). Identification of the combination of alleles at the SNP loci on the same chromosome copy, *i.e*., haplotyping, is needed to fully understand the human genetic variation patterns and enhance the power of genome-wide association studies for complex diseases [2,3]. Currently, it is expensive and labor-intensive to separate two copies of chromosomes by biological techniques [4], and most published human individuals' genomes contain only the mixed information, *i.e*., genotype information, of the underlying two copies of chromosomes [5]. Therefore, to reduce the cost, accurate and fast computational haplotyping methods are of urgent importance.

There have been many computational haplotyping models [6-8] and they can be grouped into two main classes: haplotype inference and haplotype assembly [6]. Haplotype inference is to phase the haplotypes of individuals in a pedigree or a population from their genotypes. Computer algorithms of haplotype inference have been used in the International HapMap Project [9] and the 1000 Genomes Project [5] to identify haplotypes. Haplotype assembly is also called Single Individual Haplotyping (SIH). SIH assembles a pair of haplotypes from an individual's aligned DNA sequence fragments. With the dramatically dropped cost of human whole genome sequencing, more and more human individual's DNAs have been sequenced. With mate-pairs sequencing and read length improvements of the next-generation sequencing technologies, and with the development of new sequencing technologies, SIH methods have been used to build haplotype-resolved genome of human beings [10,11]. When there are enough DNA sequence fragments that cover two or more consecutive variant loci, SIH builds longer and more accurate haplotype blocks than haplotype inference does [12].

Since Lancia *et al*., first formalized the SIH problem [13], many optimization models and algorithms have been introduced to solve the problem [7,14-25]. The main models are MEC (minimum error correction) [24], MFR (minimum fragment removal), and MSR (minimum SNP removal) [13]. Recently, Duitama *et al*., proposed a new model MFC (maximum fragments cut) [16]. Most of the models are NP-hard and APX-hard [16,21,26], and their exact algorithms run in time exponentially dependend on at least one input parameter [15,17,19-21]. Therefore, a large number of heuristic algorithms have been designed to deal with the problem [16,22,23,25]. According to [16], one of the most accurate heuristic algorithms is HapCUT [25], while ReFHap [16] runs much faster than HapCUT without loss of accuracy. In this paper, we consider both quality measures "errors corrected" and "fragments cut", and propose a new optimization model, called Balanced Optimal Partition (BOP), for the SIH problem. The model generalizes the most popular model MEC and the recent model MFC. In fact, it could be made either model by setting some parameters to extreme values. To solve the model, we propose a dynamic programming algorithm H-BOP. By limiting the number of intermediate solutions at each iteration to an appropriately chosen small integer *k*, H-BOP is able to solve the model efficiently. The time complexity of H-BOP linearly depends on some of the key parameters controlling the input size and the algorithm scales well to large input data.

## Results and discussion

We use a public available Java package SIH [27] to test the performance of H-BOP. The package contains a simulated data generator and implements algorithm ReFHap [12,16]. The simulated data are generated according to five parameters: number of SNPs (haplotype length) *n*, number of fragments *m*, average fragment length *l*, sequencing error rate *e*, and gap rate *g*. In our experiments, since we only consider heterozygous SNPs, for each data set, a haplotype *h*_1 _containing *n *SNPs is generated randomly first and then the other haplotype *h*_2 _is obtained by flipping each allele of *h*_1_. For each haplotype, *m/*2 fragments are randomly sampled from the haplotype and their lengths follow a normal distribution with mean *l *and variance 1. Finally for each fragment, every allele is flipped with probability *e *to introduce sequencing errors and, except at the first and last positions, every allele is deleted with probability *g *to introduce gaps. Given fragments generated as above, the average call coverage *c *is calculated by dividing the total number of alleles of the fragments by the haplotype length *n*. Please see [16] for more details.

Among many algorithms for the SIH problem, HapCUT and ReFHap are two of the most accurate heuristic algorithms [12,16]. Since ReFHap is much faster than HapCUT, we only compare our algorithm with ReFHap. We implemented our algorithm H-BOP in Java and embedded it in the java package SIH and tested the accuracies, phased haplotype lengths and running time of H-BOP and ReFHap on simulated data and a real data set provided by [12]. All tests are carried out on a Windows 7 64 bit PC (3GHz CPU, 4GB RAM). To measure the haplotype reconstruction accuracy of an SIH algorithm, the hamming distance between the reconstructed haplotype pair and the real haplotype pair was previously used widely in the literature [14,23]. However, a recent study [28] showed it over-penalizes simple switch errors. Therefore, as in [12,16], we use switch errors to measure the accuracy of an algorithm. A switch error is an inconsistency between the reconstructed haplotype pair and the real haplotype pair over two contiguous SNPs. There may be some SNP sites where an algorithm is unable to determine the alleles of a haplotype. The phased haplotype length is defined as the number of SNP sites where the alleles of the reconstructed haplotype pair are determined. And the number of switch errors divided by the phased haplotype length is called switch error rate.

If the allele of a fragment *f *at a SNP site *s *is known, we say *f *covers *s*. When there are no fragments covering two consecutive loci, it is not possible to determine the haplotype containing these consecutive loci for all SIH models. Therefore, for each test we divide a haplotype into blocks according to the input fragments as in [16]. A block corresponds to a connected component of a graph *G *= (*V*, *E*) where *V *is the set of the SNP sites and there is a edge between two SNP sites *s*_1 _and *s*_2 _if and only if there is a fragment covers both *s*_1 _and *s*_2_. The switch errors of an algorithm are the sum of the switch errors in all blocks. In the following simulation tests, the haplotype length *n *= 100, the gap rate *g *= 0.1 and each result is the average over 100 repeated experiments if there is no explicit specification.

### Parameters of the algorithm H-BOP

There are two parameters *w *and *k *in H-BOP. The parameter *w *is a weighting factor. H-BOP tries to seek a solution with the minimum number of errors corrected when *w *= 0, and a solution with a maximum cut of the weighted conflict graph corresponding to the input fragments [16] when *w *is set sufficiently large. *k *is the maximum number of intermediate solutions that we will keep at each iteration of H-BOP. When *k *is large enough, H-BOP in fact becomes an exact algorithm. To choose appropriate values for *w *and *k*, we test H-BOP on different combinations of *w *and *k*.

**Figure 1:**
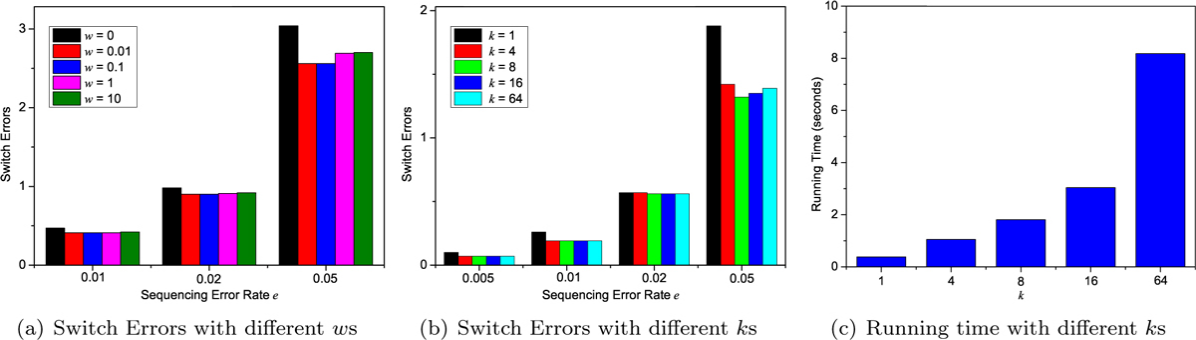
**Performances of H-BOP with different parameter values**. Here, the haplotype length *n *= 100, the gap rate *g *= 0.1 and the average fragment length *l *= 3. (a) The number of fragments *m *= 140 and *k *is 16. (b) *w *= 0.1 and *m *= 211. (c) *w *= 0.1, *m *= 211 and *e *= 0.01.

In Figure 1, the haplotype length *n *= 100 and the average fragment length *l *= 3. In Figure 1(a), the number of fragments *m *= 140 (*c *= 4.25) and *k *= 16. In Figures 1(b) and 1(c), *m *= 210 (*c *= 6.42) and *w *= 0.1. Figure 1(a) shows that when the sequencing error rate *e *increases from 0.01 to 0.05, the switch errors of H-BOP increases accordingly. The switch errors of H-BOP is larger when *w *= 0 than those when *w >*0 with *e *unchanged, which is obvious when *e *= 0.05. It indicates that the optimal objective minimum errors corrected leads large switch errors when sequencing error rate is high. When *w *= 0.01 and 0.1, the switch errors of H-BOP are smallest. Figure 1(b) shows that when *k *increases from 1 to 8, the switch errors decrease accordingly. When *k *increases from 8 to 64, there are no significant improvements in switch errors of H-BOP. When sequencing error rates are high, exact optimal solutions may incur large switch errors and Figure 1(b) indicates that when *k *increases from 8 to 64, switch errors of H-BOP increases accordingly. Figure 1(c) shows that the running time of H-BOP increases linearly with *k *when the haplotype length *n*, number of fragments *m *and average fragment length *l *remain fixed. In the following tests, we set *w *= 0.1 and *k *= 8 for H-BOP.

### Simulation results

**Figure 2:**
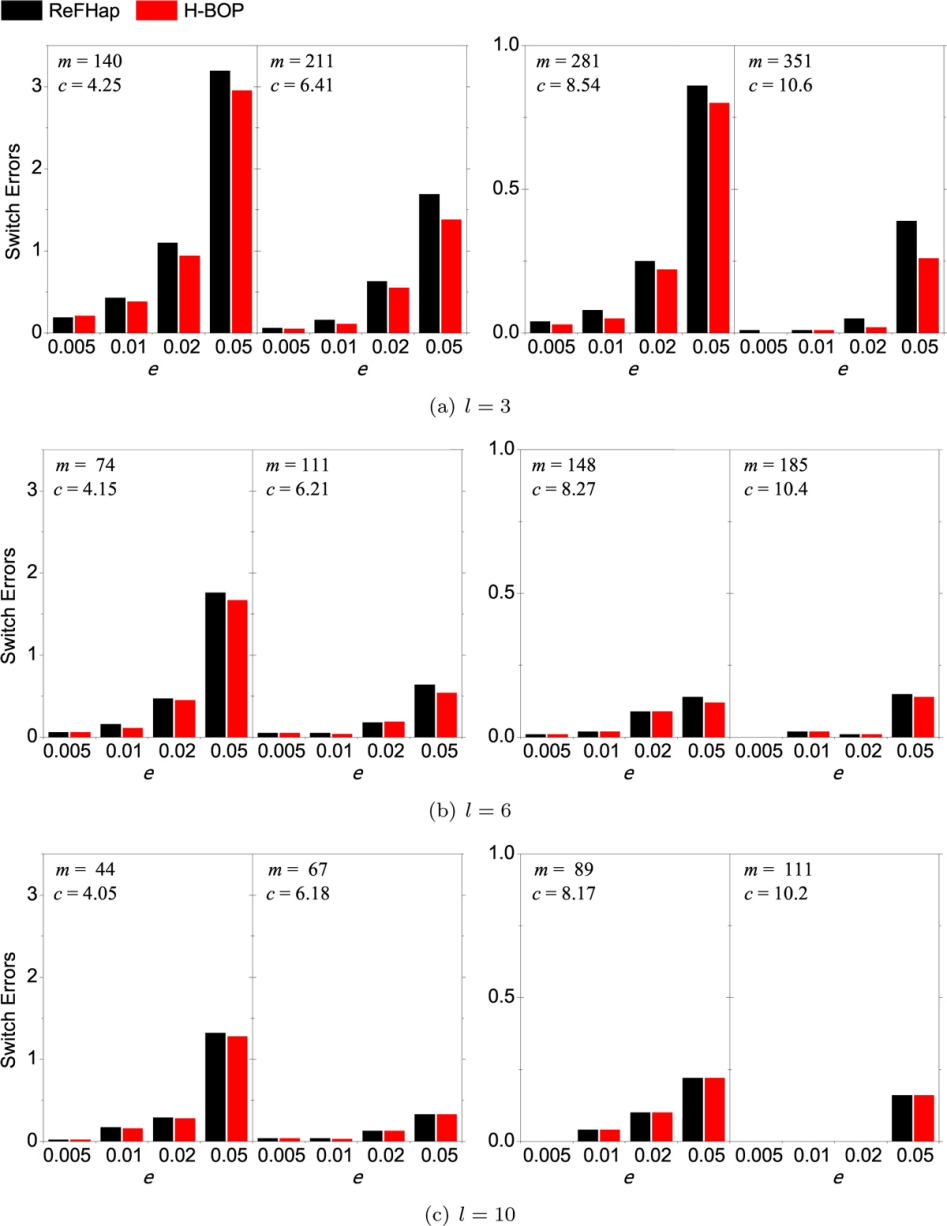
**Switch errors of ReFHap and H-BOP with varying *e*, *m *and *l***. The parameters *k *and *w *of H-BOP are set as 8 and 0.1, respectively, and the haplotype length *n *is again set as 100.

We changed the sequencing error rate *e*, the average fragment length *l *and the number of fragments *m *to generate different fragment data sets, and compared the performance of H-BOP and ReFHap. Figure 2 shows that H-BOP and ReFHap are both accurate and there are only several switch errors in reconstructing haplotypes of 100 SNPs. The accuracies of both algorithms decrease with the increase of *e *and improves with the increase of *m*. These results are consistent with those in [16], which claim that the accuracy of an SIH algorithm increases with decreasing sequencing error rate and increasing call coverage. In a half of the total 48 cases shown in Figure 2, H-BOP produces fewer switch errors than ReFHap, especially when *l *= 3 and *e *≥ 0.02. In the other half, H-BOP presents a few more switch errors than ReFHap only in two cases (*i.e*., Figure 2(a), *m *= 140, *e *= 0.005 and Figure 2(b), *m *= 111, *e *= 0.02). In the remaining 22 cases, H-BOP has the same switch errors as ReFHap.

The phased haplotype lengths of both algorithms generally increase with decreasing *e *and increasing *m*. Table 1 shows that when *l *= 3 and *m <*351, or when *e *= 0.05 (except when *l *= 10 and *m >*44), H-BOP is able to phase more SNPs than ReFHap. In other cases the phased haplotype lengths of H-BOP and ReFHap are equal.

**Table 1:** Average phased haplotype lengths of ReFHap and H-BOP

		*l *= 3				*l *= 6				*l *= 10			
			
	*m *=	140	211	281	351	74	111	148	185	44	67	89	111
*e *= .005	ReFHap	98.16	99.59	99.79	99.97	97.2	99.04	99.39	99.66	96.24	97.7	98.51	99.06
	H-BOP	98.17	99.61	99.8	99.97	97.2	99.04	99.39	99.66	96.24	97.7	98.51	99.06

*e *= .01	ReFHap	97.99	99.6	99.68	99.9	97	98.87	99.32	99.59	95.68	97.81	98.73	99.13
	H-BOP	98.07	99.63	99.7	99.9	97	98.87	99.32	99.59	95.69	97.81	98.73	99.13

*e *= .02	ReFHap	97.42	99.39	99.71	99.86	96.94	98.59	99.48	99.63	95.31	98.06	98.24	99.22
	H-BOP	97.57	99.48	99.74	99.86	96.95	98.59	99.48	99.63	95.31	98.06	98.24	99.22

*e *= .05	ReFHap	96.6	98.88	99.49	99.77	95.61	98.31	99.14	99.5	94.25	97.56	98.62	98.87
	H-BOP	96.94	99.09	99.63	99.84	95.76	98.42	99.15	99.51	94.33	97.56	98.62	98.87

The numbers in the table are the average of 100 repeated tests.

**Figure 3:**
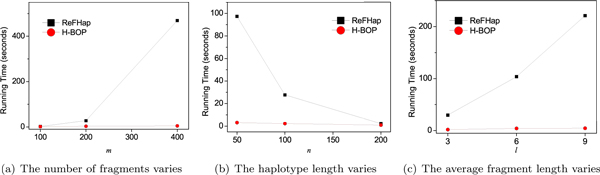
**Running time of ReFHap and H-BOP**. (a) The number of fragments *m *varies with *n *= 100 and *l *= 3. (b) The haplotype length *n *varies with *m *= 200 and *l *= 3. (c) The average fragment length *l *varies with *m *= 200 and *n *= 100.

We set *e *= 0.01 and varied the haplotype length *n*, the number of fragments *m *and the average fragment length *l *to compare the running time of H-BOP and ReFHap (Figure 3). When *n *= 100, *l *= 3 and *m *increases from 100 to 400, the running time of H-BOP increases linearly, but the running time of ReFHap increases sharply (Figure 3(a)). When *m *reaches 400, the running time of H-BOP is only about 4 seconds, while that of ReFHap is about 468 seconds. When *n *increases from 50 to 200 while *m *= 200 and *l *= 3, the average call coverage decreases and the running time of both algorithms decreases accordingly (Figure 3(b)). When *m *= 200, *n *= 100 and *l *increases from 3 to 9, the running time of ReFHap increases significantly while that of H-BOP increases slowly and remains less than 5 seconds (Figure 3(c)). When the number of fragments and the average fragment length increase, the average call coverage *c *increases accordingly. When *c *is large, H-BOP runs much faster than ReFHap.

## Results on real data

We downloaded a real data set from the SIH website [27], which contains the aligned sorted fosmid-based NGS DNA sequence fragments and gold-standard haplotypes of a HapMap trio child, NA12878 [12]. The total heterozygous SNP sites of the data are 1,704,166, the total fragments are 285,341, the average fragment length is 18.03, and the average call coverage is 3.02. Since the coverage is low, there are many consecutive heterozygous SNP site pairs not covered by any fragments, and hence the 23 pairs of chromosomes are divided into 17,839 haplotype blocks. Due to the low coverage, both H-BOP and ReFHap ran very fast and completed the reconstruction of haplotypes for all 23 pairs of chromosomes in about half a minute. The total phased haplotype lengths of H-BOP and ReFHap are 1,563,741 and 1,562,402 respectively. Compared with the gold-standard haplotypes, the total switch errors of the haplotypes built by H-BOP and ReFHap are 21,859 and 21,835 respectively. Though the switch errors of H-BOP is larger than that of ReFHap, the switch error rates of H-BOP and ReFHap are both 0.014.

To account for both completeness and quality, Duitama *et al. *[12] proposed an alternative measure QAN50 (quality adjusted N50). QAN50 is calculated as follows [12]:

(1) Break every haplotype block into the longest possible segments containing no switch errors.

(2) Calculate span (in reference base pairs) from the first phased SNP to the last phased SNP for every segment.

(3) Adjust each span by multiplying the span with phased SNPs ratio (the number of phased SNPs divided by the number of total SNPs) inside the segment (to correct for un-phased SNPs).

(4) Sort segments from the largest to the smallest adjusted span.

(5) Traverse the list and count the number of phased SNPs. When the count is more than a half of the total number of SNPs, the adjust span of the current segment is QAN50.

Clearly, algorithms with larger QAN50 values are more desirable. The QAN50 values of H-BOP and ReFHap on the above real data set are 114,261.09 and 113,831.67, respectively.

## Conclusions

Haplotyping is regarded as one of the hardest challenges in personal genome sequencing. Though some single molecule sequencing technologies have been developed, they are still too expensive and labor-consuming. Computer algorithms are widely used to reconstruct haplotypes in personal genome sequencing. SIH uses computer algorithms to build a pair of haplotypes from an individual's aligned DNA sequence fragments. There are many different combinatorial optimization models for SIH, among which MEC is the most popular and MFC is the most recent introduction [16]. In this paper, we combine the two quality measures "errors corrected" and "fragments cut" used in MEC and MFC and introduce a new model BOP. We design a heuristic dynamic programming algorithm H-BOP to solve the model. By setting appropriate parameters of the algorithm, H-BOP is accurate and fast. Extensive simulation experiments show that H-BOP is generally faster and more accurate in assembling haplotypes than a recent state-of-art algorithm ReFHap. When the average fragment length is small and sequencing error rate is relatively high, H-BOP is significantly more accurate than ReFHap. The running time of H-BOP increases linearly as the average call coverage *c *increases, and H-BOP runs much faster than ReFHap when *c *is large. The test on a real data set also shows that H-BOP is superior to ReFHap considering both completeness and quality of the reconstructed haplotypes.

## Methods

### Formulation and problem

With the input of aligned DNA sequence fragments derived from a pair of chromosomes, SIH tries to reconstruct a pair of haplotypes of their underlying chromosomes. If there are no sequencing errors and for any two consecutive (but not necessarily adjacent) heterozygous SNP loci, there is at least one fragment covering both, we can easily determine the linkage relationship between two consecutive heterozygous SNPs and thus SIH is easy. However, sequencing errors are unavoidable which makes the problem complicated. In the following, we first introduce some notations and definitions similar to those in [15,16,20,23], and then propose a new optimization model.

Since we only consider the alleles at SNP loci in the SIH problem, the input aligned fragments are encoded as an *m × n *SNP matrix *M *[15,16,25], where *m *is the number of fragments and *n *the number of SNP loci. An entry at the *i*th row and the *j*th column of *M *is denoted as *M*[*i*, *j*]. *M*[*i*, *j*] takes a value from {0, 1, *−*}, where '0' (or '1') encodes the major allele (or the minor allele, respectively) in the population and '*−*' represents an unknown allele. As in previous work [16,25], we assume that all SNP loci are heterozygous and every fragment covers at least two heterozygous SNP loci. If this is not the case, a simple preprocessing as in [22] and [29] can be used to remove homozygous loci and fragments covering only one heterozygous SNP locus. In the remainder of the section, the *i*th row of *M *is equivalent to the *i*th fragment and the *j *column of *M *is equivalent to the *j*th SNP locus without explicit specification for briefness.

Let *a*, *b *∈ {0, 1, −} and define

(1) $$c\left(a,b\right)=\left\{\begin{array}{cc} 1, & \text{if}\;a,b\neq -\text{and}\;\alpha \neq b;\\ 0, & \text{otherwise}. \end{array}\right)$$

Given an *m × n *SNP matrix *M*, let the underlying haplotype pair be $H=\left(H_{1},H_{2}\right)$. The allele at the *j*th SNP locus of *H*_1 _(or *H*_2_) is denoted as *H*_1_[*j*] (or *H*_2_[*j*], respectively). Notice that since all SNP loci of interest are heterozygous, for any *j *∈ {1, ..., *n*}, *H*_1_[*j*] *≠ H*_2_[*j*], *i.e*. when *H*_1_[*j*] = 0, *H*_2_[*j*] = 1 and when *H*_1_[*j*] = 1, *H*_2_[*j*] = 0. Let *f_i _*denote the *i*th fragment (*i.e*. the *i*th row of *M*). *c*(*M*[*i*_1_, *j*], *M*[*i*_2_, *j*]) = 1 means that $f_{i_{1}}$ and $f_{i_{2}}$*conflict *at SNP locus *j *(*i.e*. column *j*), and that if both fragments come from the same chromosome, either *M*[*i*_1_, *j*] or *M*[*i*_2_, *j*] is a sequencing error. Similarly, *c*(*M*[*i*, *j*], *H_p_*[*j*]) = 1 (*p *= 1 or 2) means that *f_i _*and *H_p _conflict *at SNP locus *j*, and that if *f_i _*comes from the chromosome with haplotype *H_p_*, *M*[*i*, *j*] must be a sequencing error.

Let $s_{c}\left(H_{p},\;f_{i}\right)=\sum_{j=1,\ldots ,n}c\left(M\left[i,\;j\right],\;H_{p}\left[j\right]\right)$ and $s_{c}\left(H,\;f_{i}\right)=\text{min}\left(s_{c}\left(H_{1},\;f_{i}\right),\;s_{c}\left(H_{2},\;f_{i}\right)\right)$ and define

(2) $$s_{c}\left(H,\;M\right)=\underset{i=1,\ldots ,m}{\sum }\left(s_{c}\left(H,\;f_{i}\right)\right).$$

If the real haplotype pair is H , it is easy to verify that the number of sequencing errors in the input fragments is at least $s_{c}\left(H,\;M\right)$, which we call the *errors corrected measure*. Based on the principle of parsimony, it is a natural optimization objective that to minimize the number of errors and hence Minimum Error Correction [20,23,24] is the most popular model for SIH.

**Minimum Error Correction (MEC)**: Given an *m × n *SNP matrix *M*, find a haplotype pair H  such that $s_{c}\left(H,\;M\right)$ is minimized.

Based on the underlying haplotype pair $H=\left(H_{1},H_{2}\right)$, it is easy to partition the fragments into two groups *G*_1_, *G*_2 _according to the following rule: For each fragment *f_i_*, if *s_c_*(*H*_1_, *f_i_*) *< s_c_*(*H*_2_, *f_i_*), add *f_i _*to *G*_1_, otherwise add *f_i _*to *G*_2_. Let ${\mathbb{P}}_{H}$ denote the partition (*G*_1_, *G*_2_) obtained by the above rule. A partition $P=\left(G_{1},\;G_{2}\right)$ of a fragment set *R *is encoded as a map such that P(f)=0 (or 1) if the fragment *f *in *R *belongs to *G*_1 _(or *G*_2_, respectively).

Conversely, given a partition $P=\left(G_{1},\;G_{2}\right)$, a haplotype pair $H=\left(H_{1},H_{2}\right)$ can be constructed as follows. Let *N_g,v_*[*j*] be the number of fragments of *G_g _*whose allele at the *j*th locus is *v *for *g *= 1, 2 and *v *= 0, 1. For each SNP locus *j*, if *N*_1,1_[*j*] + *N*_2,0_[*j*] *≤ N*_1,0_[*j*] + *N*_2,1_[*j*], *H*_1_[*j*] = 0 and *H*_2_[*j*] = 1; otherwise, *H*_1_[*j*] = 1 and *H*_2_[*j*] = 0. Let ${ℍ}_{P}$ denote the haplotype pair obtained by the above method.

**Theorem 1 ***Given an SNP matrix M and a haplotype pair $H,s_{c}\left(H,\;M\right)\geq s_{c}\left({ℍ}_{{\mathbb{P}}_{H}},\;M\right)$*.

There is another formulation of MEC equivalent to the above one: Given an SNP matrix *M*, find a partition P  of the rows in *M *such that $s_{c}\left({ℍ}_{P},M\right)$ is minimized. In the following, $s_{c}\left({ℍ}_{P},M\right)$ is called the *errors corrected measure of *P . While MEC aims to find a partition such that the conflict between the fragments in the same group is minimized, Maximum Fragments Cut (MFC) [16] aims to find a partition such that the conflict between the fragments in *G*_1 _and the fragments in *G*_2 _is maximized.

Let *a*, *b *∈ {0, 1, *−*} and define

(3) $$d\left(a,\;b\right)=\left\{\begin{array}{cc} -1, & \text{if}\;a,\;b\neq \;-\text{and}\;a=b;\\ 1, & \text{if}\;a,\;b\neq \;-\text{and}\;a\neq b;\\ 0, & \text{otherwise}. \end{array}\right)$$

Let $f_{i_{1}}$ and $f_{i_{2}}$ be two rows of *M *and define $d\left(f_{i_{1}},\;f_{i_{2}}\right)=\sum_{j=1,\ldots ,n}\left(d\left(M\left[i_{1},\;j\right],\;M\left[i_{2},\;j\right]\right)\right)$. As in [16], we convert an *m × n *SNP matrix *M *into a weighted complete graph G=(V,E), where *V *is the set of rows in *M *and the weight of the edge between two rows $f_{i_{1}}$ and $f_{i_{2}}$ is $d\left(f_{i_{1}},f_{i_{2}}\right)$. Therefore, a partition P  of the rows in *M *corresponds a cut of G . Given an SNP matrix *M *and a partition P , the *fragments cut measure *is defined as:

(4) $$s_{d}\left(P,\;M\right)=\underset{P\left(f_{i_{1}}\right)=1,P\left(f_{i_{2}}\right)=2}{\sum }d\left(f_{i_{1}},\;f_{i_{2}}\right).$$

**Maximum Fragments Cut (MFC) **[16]: Given an SNP matrix *M*, find a partition P  of the rows of *M *such that $s_{d}\left(P,M\right)$ is maximized.

To take into account both the conflict between the two groups and the conflict between the fragments within the same group, we introduce a new score combining the above errors corrected measure and the fragments cut measure. Given a partition P  of the rows of *M*, define the *partition score *as

(5) $$s_{p}\left(P,\;M\right)=s_{c}\left({ℍ}_{P},\;M\right)-ws_{d}\left(P,\;M\right),$$

where *w *is a weight factor that is used to adjust the weight of the fragments cut measure. In the following we propose a new optimization model for the SIH problem.

**Balanced Optimal Partition (BOP)**: Given an SNP matrix *M*, find a partition P  of the rows in *M *such that $s_{p}\left(P,M\right)$ is minimized. A solution to *BOP *of *M *is denoted by *BOP(M)*, *i.e*. a partition with the minimum partition score.

Note that when *w *= 0, BOP becomes MEC which has been proved NP-hard [24] and APX-hard [26]. Therefore, BOP is NP-hard and APX-hard.

### Algorithm

Given an *m × n *SNP matrix *M*, there are 2^*m-*1 ^different partitions of *m *rows in *M*. Therefore, when *m *is large, it is impractical to enumerate all possible partitions and choose one with the minimum partition score. To solve the BOP model of *M *efficiently, we propose a dynamic programming algorithm in the subsection. We first consider the first row of *M*, then the first two rows and so on until we have considered all rows of *M*.

We need some definitions and notations. Let *M *[1..*i*, :] denote the SNP matrix consisting of only the first *i *rows of *M*. The first and last columns at which the *i*th row of *M *takes non '*−*' values are denoted by *l*(*i*) and *r*(*i*), respectively. For a column *j*, if *l*(*i*) *≤ j ≤ r*(*i*), row *i spans *column *j*. In the following, we assume that all the rows of *M *are sorted such that if *i*_1 _*< i*_2_, *l*(*i*_1_) *< l*(*i*_2_) or *l*(*i*_1_) = *l*(*i*_2_) ∧ *r*(*i*_1_) *≤ r*(*i*_2_). Let *R*(*i*) denote the row set containing the rows in *M*[1..*i*, :] that span column *l*(*i*).

Let P  be a partition of a row set *R *and $P^{\prime }$ a partition of a subset $R^{\prime }$ of *R*. If for every row *i *∈ *R*', $P\left(i\right)=P^{\prime }\left(i\right)$, $P^{\prime }$ is called the *projection *of P  on *R*' and P  is called an *extension *of $P^{\prime }$ on *R*. Fix a row *i *and let P  be a partition of *R*(*i*). $P^{\prime }$ is an *optimal extension *of P , if the following conditions hold: (1) $P^{\prime }$ is an extension of P  on the row set *R *= {1, ..., *i*}; (2) for any possible extension $P^{\prime \prime }$ of P  on *R*, $s_{p}\left(P^{\prime },\;M\left[1..i,\;:\right]\right)\leq s_{p}\left(P^{\prime \prime },\;M\left[1..i,\;:\right]\right)$.

Given a partition P  of *R*(*i*), let $\varepsilon_{i}\left(P\right)$ denote an optimal extension of P . We call $s_{p}\left(E_{i}\left(P\right),\;M\left[\text{l}..i,\;:\right]\right)$ the partition score of P  and denote it as $s_{p}^{i}\left(P\right)$ for briefness.

**Theorem 2 ***For an m *× *n SNP matrix M, let *P *be a partition of *$R\left(m\right).\;E_{m}\left(P\right)$ is a solution to BOP of M if the following condition holds: for any possible partition $P^{\prime }$*of R*(*i*), $s_{p}^{m}\left(P\right)\leq s_{p}^{m}\left(P^{\prime }\right)$.

Consider the submatrix containing only the first row of *M*. Since *R*(1) contains only one row, *i.e. R*(1) = {1}, there are only two possible partitions $P_{1}$ and $P_{2}$ of *R*(1) ($P_{1}$ and $P_{2}$ are in fact equivalent): $P_{1}\left(1\right)=0$ iff $P_{2}\left(1\right)=1$. It is easy to verify that the following equalities hold for *i *= 1*; *2.

(6) $$E_{1}\left(P_{i}\right)=P_{i};s_{p}^{1}\left(P_{i}\right)=0.$$

After $E_{i}\left(P\right)$ and $s_{p}^{i}\left(P\right)$ have been calculated for every possible partition P  of *R*(*i*), we consider the submatrix containing the first *i *+ 1 rows of *M*. Let *R_c_*(*i*, *i *+ 1) = *R*(*i*) *∩ R*(*i *+ 1), and we calculate $E_{i+1}\left(P^{\prime }\right)$ and $s_{p}^{i+1}\left(P^{\prime }\right)$ for every possible partition $P^{\prime }$ of *R*(*i *+ 1) according to the following method. Let *q *be the number of the rows in *R*(*i*) but not in *R_c_*(*i*, *i *+ 1), *i.e. q *= *|R*(*i*)*−R_c_*(*i*, *i *+ 1)*|*. For a partition $P^{\prime \prime }$ of *R_c_*(*i*, *i *+ 1), there are 2*^q ^*distinct extensions of $P^{\prime \prime }$ on *R*(*i*). Suppose $P_{m}$ is the one whose partition score is the minimum among all 2*^q ^*extensions. Then $E_{i}\left(P^{\prime \prime }\right)$ and $s_{p}^{i}\left(P^{\prime \prime }\right)$ can be computed with the following equations:

(7) $$E_{i}\left(P^{\prime \prime }\right)=E_{i}\left(P_{m}\right);s_{p}^{i}\left(P^{\prime \prime }\right)=s_{p}^{i}\left(P_{m}\right).$$

Since the rows in *M *are sorted, it is easy to verify that *R*(*i *+ 1) = *R_c_*(*i*, *i *+ 1) ∪ {*i *+ 1}, and that the number of all possible distinct partitions of *R*(*i *+ 1) is two times that of *R_c_*(*i*, *i *+ 1). For each partition $P^{\prime \prime }$ of *R_c_*(*i*, *i *+ 1), there are two distinct corresponding partitions ${P_{1}}^{\prime }$ and ${P_{2}}^{\prime }$ of *R*(*i *+ 1): for each *l *∈ *R_c_*(*i*, *i *+ 1), ${P_{1}}^{\prime }\left(l\right)={P_{2}}^{\prime }\left(l\right)=P^{\prime \prime }\left(l\right);{P_{1}}^{\prime }\left(i\right)=0$, but ${P_{2}}^{\prime }\left(i\right)=1$. Optimal extensions of ${P_{1}}^{\prime }$ and ${P_{2}}^{\prime }$ and their partition scores can be calculated with the following equations:

(8) $$E_{i+1}\left({P_{1}}^{\prime }\right)\left(l\right)=\left\{\begin{array}{cc} E_{i}\left(P^{\prime \prime }\right)\left(l\right), & 1\leq l<i;\\ {P^{\prime }}_{1}\;\left(l\right), & l=i. \end{array}\right)$$

(9) $$E_{i+1}\left({P_{2}}^{\prime }\right)\left(l\right)=\left\{\begin{array}{cc} E_{i}\left(P^{\prime \prime }\right)\left(l\right), & 1\leq l<i;\\ {P^{\prime }}_{2}\;\left(l\right), & l=i. \end{array}\right)$$

(10) $$s_{p}^{i+1}\left({P_{1}}^{\prime }\right)=s_{p}^{i}\left(P^{\prime \prime }\right)+\delta_{c}\left({P_{1}}^{\prime }\right)-w\delta_{d}\left({P_{1}}^{\prime }\right);$$

(11) $$s_{p}^{i+1}\left({P_{2}}^{\prime }\right)=s_{p}^{i}\left(P^{\prime \prime }\right)+\delta_{c}\left({P_{2}}^{\prime }\right)-w\delta_{d}\left({P_{2}}^{\prime }\right).$$

In Equation (10) (or 11), $\delta_{c}\left({P_{1}}^{\prime }\right)\;\left(\text{or }\delta_{c}\left({P_{2}}^{\prime }\right)\right)$ is the difference between the errors corrected measures of $E_{i+1}\left({P_{1}}^{\prime }\right)\;\left(\text{or }E_{i+1}\left({P_{2}}^{\prime }\right)\right)$ and $E_{i}\left(P^{\prime \prime }\right)$; and $\delta_{d}\left({P_{1}}^{\prime }\right)\;\left(\text{or }\delta_{d}\left({P_{2}}^{\prime }\right)\right)$ is the difference between the fragments cut measures of $E_{i+1}\left({P_{1}}^{\prime }\right)\;\left(\text{or }E_{i+1}\left({P_{2}}^{\prime }\right)\right)$ and $E_{i}\left(P^{\prime \prime }\right)$. The values $\delta_{c}\left(P\right)$ and $\delta_{d}\left(P\right)$ are calculated by calling the following functions DeltaEC(i,P) and DeltaFC(i,P), respectively.

   DeltaEC(i,P)

    { *δ *= 0;

      **for ***j *= *l*(*i *+ 1),..., *r*(*i *+ 1) **do**

      **{ if ***M*[*i *+ 1, *j*] = = '-' **then **continue;

         *N*_1,0 _= *N*_2,0 _= *N*_1,1 _= *N*_2,1 _= 0;

         **for **each row *l *∈ *R_c_*(*i*, *i *+ 1) **do**

         { **if ***M*[*l*, *j*] = = '-' **then **continue;

            *v *= *M*[*l*, *j*], *g *= $P\left(l\right)$,*N_g,v _*+ +; }

         **if ***N*_1,1 _+ *N*_2,0 _≤ *N*_1,0 _+ *N*_2,1 _**then**

         { *δ *= *δ - *(*N*_1,1 _+ *N*_2,0)_; }

         **else **{ *δ *= *δ - *(*N*_1,0 _+ *N*_2,1)_; }

         *v *= *M*[*i *+ 1, *j*], *g *= $P\left(i+1\right)$, *N*_*g,v *_+ +;

         **if ***N*_1,1 _+ *N*_2,0 _≤ *N*_1,0 _+ *N*_2,1 _**then**

         { *δ *= *δ *+ (*N*_1,1 _+ *N*_2,0_); }

         **else **{ *δ *= *δ *+ (*N*_1,0 _+ *N*_2,1_); }

      }

      **return ***δ*; }

   DeltaFC(i,P)

   { *δ *= 0, *g*_0 _= $P\left(i+1\right)$;

      **for ***j *= *l*(*i *+ 1), ..., *r*(*i *+ 1) **do**

      **{ if ***M*[*i *+ 1, *j*] = = '-' **then **continue;

         **for **each row *l *∈ *R_c_*(*i*, *i *+ 1) **do**

         **{ if ***M*[*l*, *j*] = = '-' **then **continue;

            *v *= *M*[*l*, *j*], *g *= $P\left(l\right)$;

            **if ***g *== *g*_0 _**then **continue;

            **if ***v *== *M*[*i *+ 1, *j*] **then ***δ - -*;

      **else ***δ *+ +; }

   }

   **return ***δ*; }

When $E_{m}\left(P\right)$ and $s_{p}^{m}\left(P\right)$ are known for every possible partition P  of *R*(*m*), a solution to BOP of *M *is easily obtained by using the following formula:

(12) $$BOP\left(M\right)=E_{m}\left(P\right)|s_{p}^{m}\left(P\right)\;\text{is minimum}.$$

**Figure 4:**
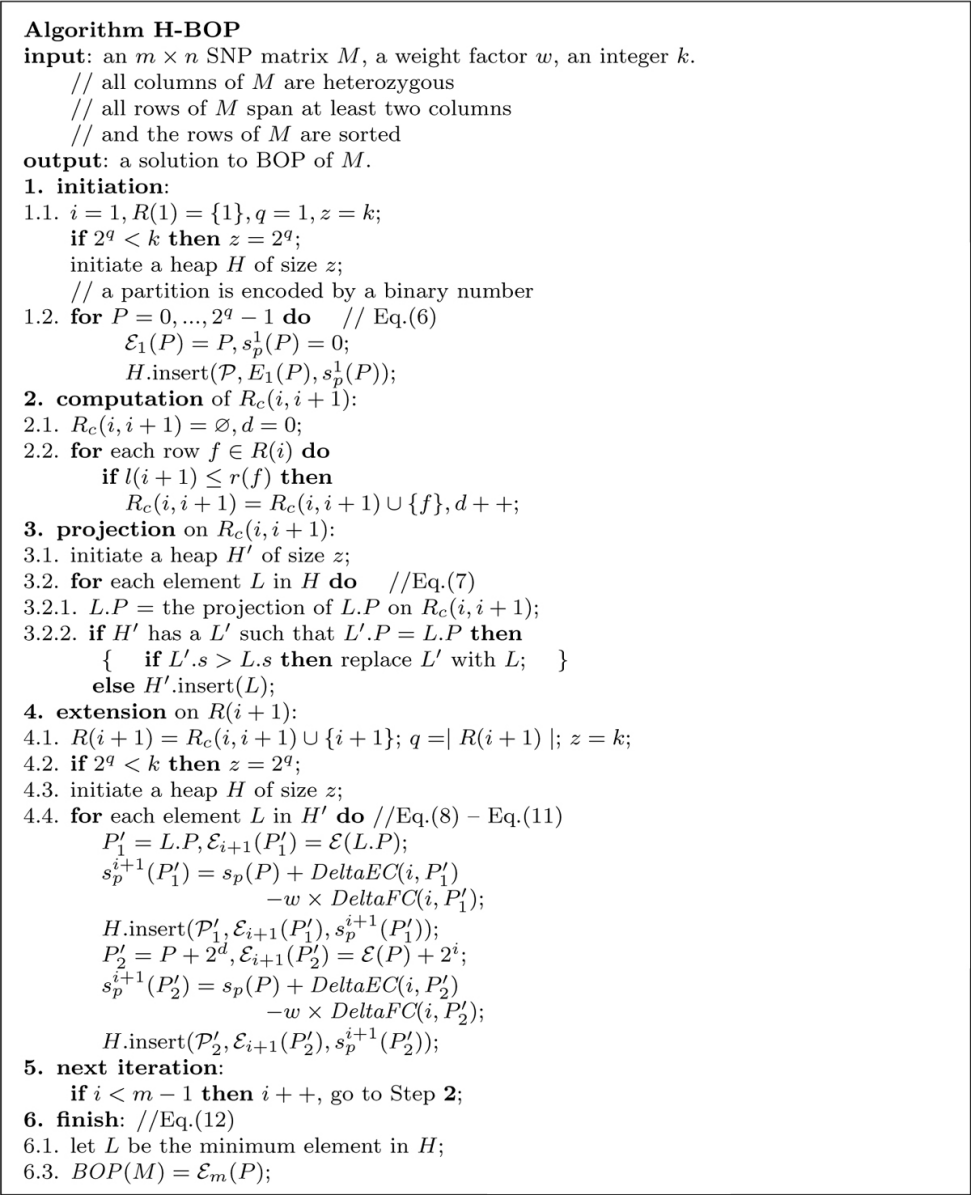
**H-BOP Algorithm**.

Based on the above equations, we can construct an exact dynamic programming algorithm for BOP. However, the complexity of this exact algorithm increases exponentially with the number of rows in *R*(*i*), which implies that the algorithm is impractical when the call coverage is large. Let $P_{i}^{*}$ be the projection on *R*(*i*) of the global optimal partition of *M*. If the partition score of $P_{i}^{*}$ is among the *k *smallest ones of all possible partitions of *R*(*i*), we only need to compute *k *partitions of *R*(*i*) whose partition scores are the smallest in each iteration without losing the global optimal partition at the end. Based on the above idea, we propose a heuristic algorithm H-BOP whose pseudo-code is shown in Figure 4. In the algorithm, a partition P  of a row set is encoded by a binary number *P*, and P(i) is represented by the *i*th bit of *P*. Therefore, the number set {0, ..., 2*^q ^− *1} encodes all possible partitions of a row set containing *q *rows (in fact, there are only 2^*q*-1 ^different partitions, and in our implement of H-BOP, we use a binary number of *q − *1 bits to encode a partition to save time and space). In each iteration of H-BOP, for each row *i *we maintain a binary max heap *H *to store the candidate partitions of *R*(*i*) whose partition scores are among the *k *smallest. The heap *H *can store at most *k *elements, and each element *L *of *H *contains a partition P , $E_{i}\left(P\right)$ and $s_{p}^{i}\left(P\right)$, which are denoted as *L.P*, *L*. E , and *L.s *respectively. The value $s_{p}^{i}\left(P\right)$ of each element in the heap is larger than or equal to those of its two children. When the number of elements of *H *is smaller than its expected size (*i.e. k*), and a new element *L *arrives, the element is inserted into *H *and *H *is adjusted to maintain the max heap property. When the number of elements of *H *reaches *k*, *L *is compared with the root *r *of *H*. If *L*.*s < r.s*, the root is replaced by *L *and *H *is adjusted accordingly; otherwise, the new element is discarded. The above operation is denoted by H.insert(*L*).

The time complexity of H-BOP is *O*(*mkk*_1_*k*_2_), and the space complexity is *O*(*mk*_1 _+ *mk*), where $k_{1}=\underset{i=1,\ldots ,m}{\text{max}}\left(r\left(i\right)-l\left(i\right)+1\right)$ and $k_{2}=\underset{i=1,\ldots ,m}{\text{max}}\left(\left|R\left(i\right)\right|\right)$.

## Competing interests

The authors declare that they have no competing interests.

## Authors' contributions

MX designed the algorithm, performed the computational experiments, and drafted the manuscript. TJ and JW helped to draft and polish the manuscript. All authors read and approved the manuscript.
